# Biomarkers for prediction of CAR T therapy outcomes: current and future perspectives

**DOI:** 10.3389/fimmu.2024.1378944

**Published:** 2024-03-15

**Authors:** Lucija Levstek, Larisa Janžič, Alojz Ihan, Andreja Nataša Kopitar

**Affiliations:** Institute of Microbiology and Immunology, Faculty of Medicine, University of Ljubljana, Ljubljana, Slovenia

**Keywords:** CAR T cells, adoptive cell immunotherapy, predictive biomarkers, therapeutic response, cytokine release syndrome, immune effector cell-associated neurotoxicity syndrome

## Abstract

Chimeric antigen receptor (CAR) T cell therapy holds enormous potential for the treatment of hematologic malignancies. Despite its benefits, it is still used as a second line of therapy, mainly because of its severe side effects and patient unresponsiveness. Numerous researchers worldwide have attempted to identify effective predictive biomarkers for early prediction of treatment outcomes and adverse effects in CAR T cell therapy, albeit so far only with limited success. This review provides a comprehensive overview of the current state of predictive biomarkers. Although existing predictive metrics correlate to some extent with treatment outcomes, they fail to encapsulate the complexity of the immune system dynamics. The aim of this review is to identify six major groups of predictive biomarkers and propose their use in developing improved and efficient prediction models. These groups include changes in mitochondrial dynamics, endothelial activation, central nervous system impairment, immune system markers, extracellular vesicles, and the inhibitory tumor microenvironment. A comprehensive understanding of the multiple factors that influence therapeutic efficacy has the potential to significantly improve the course of CAR T cell therapy and patient care, thereby making this advanced immunotherapy more appealing and the course of therapy more convenient and favorable for patients.

## 1 Introduction

Chimeric antigen receptor (CAR) T cell therapy holds enormous potential for the treatment of hematologic malignancies and shows promise for solid tumors treatment as well. This innovative approach involves reprogramming patient’s T cells to recognize and attack cancer cells through engineered receptors known as CARs. As research and clinical applications evolve, CAR T cell therapies have been developed across multiple generations, each with distinct features aimed at enhancing therapeutic efficacy and safety. The first generation of CAR T cells laid the groundwork by introducing a singular signaling domain, typically CD3ζ, to activate T cells upon antigen recognition. However, their clinical impact was limited due to modest T cell proliferation and persistence (1). Second-generation CAR T cells improved upon this by incorporating an additional costimulatory domain (such as CD28 or 4-1BB) alongside CD3ζ. This enhancement significantly boosted T cell expansion, lifespan, and antitumor activity, representing a leap forward in therapeutic effectiveness (2). Third-generation CARs further advanced the design by including two costimulatory domains, aiming to amplify T cell activation and antitumor responses even more (2). The fourth generation, often referred to as TRUCKs (T cells redirected for universal cytokine killing), are engineered to secrete proinflammatory cytokines upon engaging with tumor antigens. This feature is intended to recruit additional immune effector cells to the tumor site, intensifying the immune response (3). The fifth-generation CAR T cells, which incorporate novel signaling domains, are designed to mimic the complete activation pathway of natural T cells, offering the promise of even more potent and selective cancer targeting capabilities (4).

Despite their potential, CAR T cell therapies are associated with significant adverse events. Cytokine release syndrome (CRS) is often considered the most common side effect of CAR T cell therapy, which results from the massive release of cytokines by activated T cells and other immune cells. Symptoms can range from mild flu-like symptoms, such as fever, fatigue, and myalgia, to severe life-threatening conditions, including hypotension, high fever, and multi-organ dysfunction (5). Immune effector cell-associated neurotoxicity syndrome (ICANS) is another common side effect of CAR T cell therapy, characteristic of a wide range of neurological symptoms. These can include headache, confusion, aphasia, tremors, seizures, and in severe cases, cerebral edema (6). Other common side effects include B-cell aplasia, off-tumor cytotoxicity, tumor lysis syndrome (TLS), macrophage activation syndrome (MAS), and other less frequent adverse events (7, 8).

Despite the benefits of this promising treatment approach, it is still used as a second line of therapy for patients who relapsed after at least two previous lines of cancer therapy, or for whom for any reason other therapies can no longer be considered effective (9). The limitations of CAR T cell therapy arise primarily from severe side effects during treatment course, mainly CRS and ICANS, which can result in multiple organ dysfunction and even death. Overview of incidence of CRS and ICANS and their severity in patients treated with CAR T cell therapies is shown in **Table 1**. Accurate monitoring and efficient response times for intervention after the onset of side effect symptoms are seldom achieved because side effect symptoms usually occur rapidly and share many similarities with the regular therapy progression (inflammation, fever, fatigue, confusion, nausea, headache, rapid heart rate, etc.). Another substantial challenge in the field of CAR T cell therapy lies in addressing the issue of patient unresponsiveness. It has been observed that up to 36% of patients eligible for CAR T cell therapy undergo treatment, only to be later identified as non-responders (29). For these non-responders, the aftermath of an unsuccessful CAR T treatment can be particularly dire; it often becomes too late to pursue alternative treatments, leading to deteriorating outcomes or even death. This predicament necessitates significant research aimed at identifying potential non-responders prior to initiating CAR T cell therapy. This would enable these patients to be redirected toward alternative, more appropriate cancer therapies. Furthermore, it has the potential to alleviate the financial burden associated with unsuccessful treatment attempts. Given that the cost of CAR T therapy can range from 50,000 to several hundred thousand euros, its ineffectiveness in non-responders represents not only a therapeutic failure but also a substantial economic setback. Hence, efforts to preemptively distinguish responders from non-responders could significantly improve the cost-effectiveness and overall success rate of this innovative treatment approach. The therapy exploits the patient’s own immune system as a tool to fight cancer and, due to the heterogeneous immune traits of each individual, more personalized approaches are needed to improve therapeutic outcomes and patient care. In order to improve therapeutic efficacy, it is necessary to develop better biomarker models for predicting immune system response to CAR T cell infusion, cytotoxic efficacy of the infusion product, side effect susceptibility of each patient, therapeutic outcomes, and long-term remission.

**Table 1:** Overview of incidence of CRS and ICANS and their severity in patients treated with CAR T cell therapies.

Target Antigen	N	CR (%)	CRS (%)	Severe CRS* (%)	ICANS (%)	Severe ICANS* (%)	Ref.
ALL							
CD19	30	90	100	27	43	NA	(10)
	75	81	77	46	40	13	(11)
	53	83	85	26	44	42	(12)
	43	93	93	23	49	21	(13)
	35	69	94	17	40	6	(14)
**Average:**		**83**	**90**	**28**	**43**	**21**	
NHL							
CD19	32	34	63	13	28	28	(15)
	28	57	57	18	39	11	(16)
	101	54	93	13	64	28	(17)
	111	40	58	22	21	12	(18)
	269	53	42	2	30	10	(19)
**Average:**		**48**	**63**	**14**	**36**	**18**	
CLL							
CD19	14	29	64	43	43	7	(20)
	24	21	83	8	33	25	(21)
	38	28	63	24	8	0	(22)
**Average:**		**26**	**70**	**25**	**28**	**11**	
MM							
BCMA	16	63	94	38	NA	NA	(23)
	57	68	90	7	2	0	(24)
	25	8	88	32	32	12	(25)
	33	45	76	6	42	3	(26)
	128	33	84	5	18	3	(27)
**Average:**		**43**	**86**	**18**	**24**	**5**	
MCL							
CD19	68	67	91	15	63	31	(28)

N, Number of patients; CR, Complete response; CRS, Cytokine release syndrome; ICANS, Immune effector cell-associated neurotoxicity syndrome; ALL, B-cell acute lymphoblastic leukemia; NHL, Non-Hodgkin lymphoma; CLL, B-cell chronic lymphocytic leukemia; MM, Multiple myeloma; MCL, Mantle cell lymphoma.

*Grade 2-4. NA, Not analysed.

Numerous researchers worldwide have sought to identify effective predictive biomarkers, albeit so far only with limited success. The Eastern Cooperative Oncology Group (ECOG) performance status is a general scale used to evaluate disease progression and the patient’s abilities in daily living (30, 31). Considerable attention has been paid to estimating tumor burden prior to CAR T cell therapy, as lower tumor burden and biomass are preferred for an effective antitumor response by CAR T cells. Although tumor burden is a critical factor influencing the success of CAR T therapy (12, 32, 33), the presence of disseminated tumor already serves as a primary exclusion criterion for this treatment. Some researchers propose that assessing the tumor burden prior to CAR T cell therapy may predict therapy’s outcome (33–35). However, given the stringent inclusion criteria and the complex mechanisms affecting the therapy’s outcome and the onset of side effects, this strategy alone is not comprehensive enough for effective prediction of therapy progression (36). Clinical evidence also suggests that ≥ 3 prior lines of therapy may predict inferior survival, suggesting that CAR T therapy may be more effective if given earlier (37).

Another commonly used predictive model is the CAR-HEMATOTOX score, which captures cytopenias (thrombocytopenia, anemia, neutropenia, etc.) and inflammatory markers (C-reactive protein (CRP), ferritin, etc.) at baseline condition (38, 39). Factors included in the CAR-HEMATOTOX score are associated with prolonged cytopenias following CAR T cell therapy (38). Even though studies cite that CAR-HEMATOTOX score represents an easy-to-use risk-stratification tool that is helpful in ruling out patients at risk of hematotoxicity, the baseline CAR-HEMATOTOX score alone did not prove to be an accurate predictor of CAR T therapy progression (39, 40).

The Inflammation-Based Prognostic Score (IBPS) is a validated approach assessing systemic immune inflammation as well as a prognostic nutritional index which might prove useful in predicting CAR T therapy outcomes, however, further research is needed (41). Furthermore, the Endothelial Activation and Stress Index (EASIX) score, a marker of endothelial damage, was tested to predict the occurrence of CAR T therapy side effects. However, the major limitation of the EASIX score arises from the use of surrogate blood biomarkers that do not directly indicate endothelial damage but could also be associated with other pathologic conditions. The EASIX score is based on baseline blood levels of lactate dehydrogenase (LDH), creatinine, platelets, and additionally CRP and ferritin (42–44).

Another prediction score called the modified Cumulative Illness Rating Scale (CIRS), is used to assess comorbidities in patients with hematologic malignancies. The comorbidities with the highest impact on therapy prognosis have been classified into four main categories, referred to as the ˝Severe4˝ (encompassing the respiratory, upper gastrointestinal, hepatic, and renal systems). Patients with an overall CIRS score ≥ 7 before CAR T cell therapy, indicating severe or life-threatening comorbidities, were associated with worse CAR T therapy progression and overall survival (37, 45, 46). Although severe comorbidities serve as a prediction of poor therapy response, not many patients bear other severe illnesses. Therefore, the CIRS score is only useful for distinguishing between therapy responders and non-responders in this small group of critically ill patients, but not in patients without comorbidities or for identifying patients at increased risk for developing severe side effects (47).

Other studies have demonstrated statistically significant correlations of specific single biomarkers (e.g., LDH, programmed cell death protein 1 (PD-1), ferritin, CRP, interleukin 6 (IL-6), interleukin 15 (IL-15), etc.) with therapy progression prior to CAR T cell infusion, but failed to encapsulate the complexity of the immunologic response to CAR T cells and their antitumor effect (48–53). While many of the aforementioned prediction scores show correlations with CAR T therapy outcomes and the occurrence of adverse effects, they are unable to capture the intricate combinations of various factors involved in the antitumor activity of the infused CAR T cells and the immune system response. Therefore, more robust and complex prediction scores are needed.

The aim of this review is to identify six principal groups of predictive biomarkers and propose their use in the development of improved and efficient models for early prediction of outcomes and adverse effects in CAR T cell therapy. This approach captures various aspects of the immune response, which is a critical factor in developing robust predictive models intended for a broader population. Our review focuses on potential blood markers that can be measured using common methods, as well as advanced immunological techniques. The main focus is on markers where even minor changes in blood concentrations could have a significant value in accurately predicting the therapy progression. This is an innovative new concept that has never been explored before into such detail. It has the potential to significantly improve the course of CAR T cell therapy and patient care, thereby making this advanced immunotherapy more appealing and the course of therapy more convenient and favorable for patients.

## 2 Prospective groups of biomarkers to predict progression of CAR T cell therapy

### 2.1 Changes in mitochondrial dynamics

**Figure 1:**
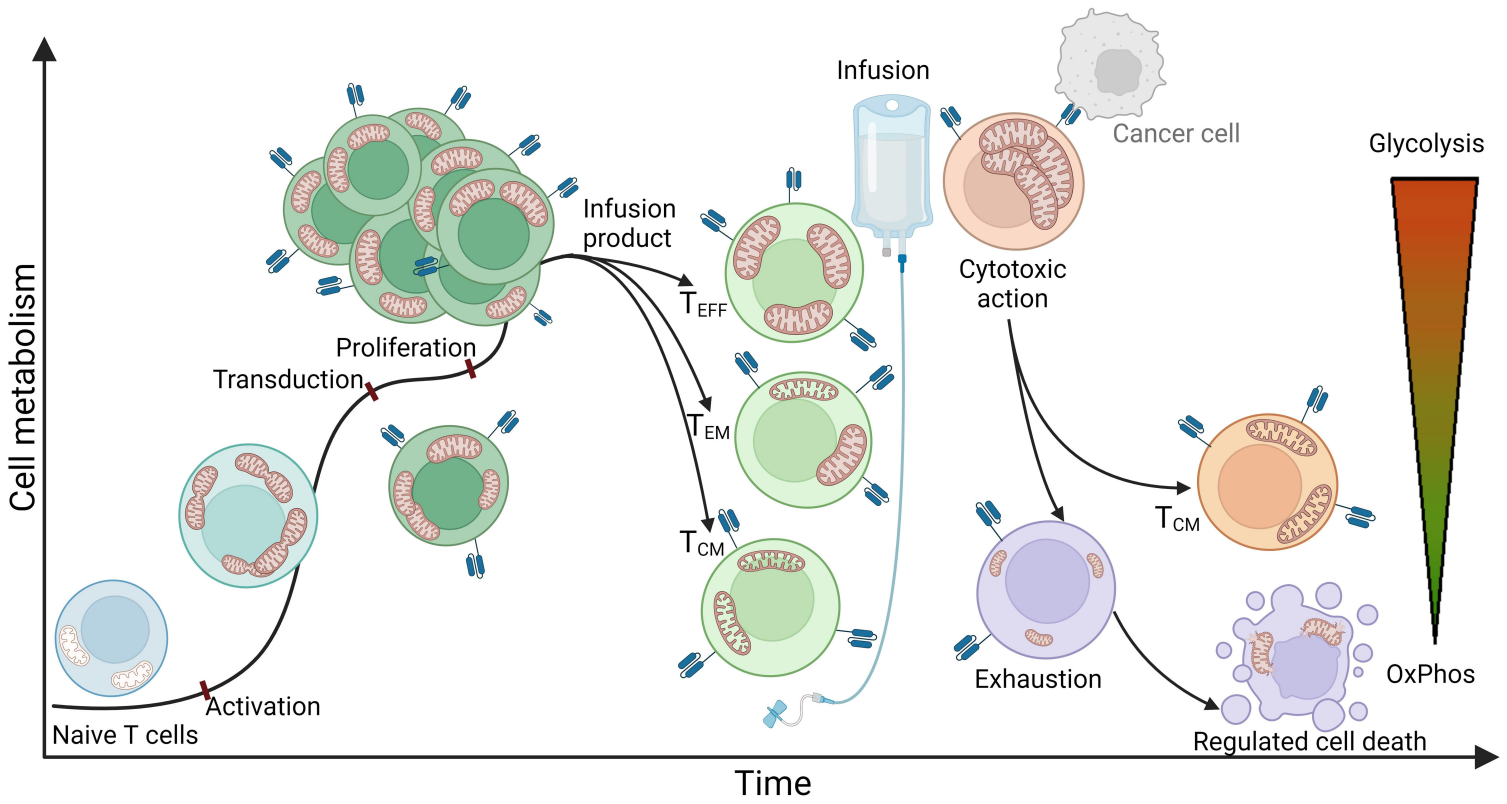
An idealized representation of T cell metabolism and mitochondrial dynamics in CAR T cell therapy. The figure illustrates a simplified representation of T cell metabolism and mitochondrial dynamics during the stages of CAR T cell therapy. The process begins with naive T cells characterized by quiescent mitochondria that mainly use oxidative phosphorylation (OxPhos) as a metabolic pathway. The obtained T cells are transferred to the CAR T production process, where they first undergo activation. This stage is characterized by a significant increase in energy demand and consequently a shift in metabolism towards glycolysis. At the same time, mitochondria undergo fission, multiplication, and formation of cristae - intricate invaginations of the inner membrane that serve to expand the surface area of the inner membrane to increase energy production capacity. After activation, the cells are genetically modified, usually by exploiting viral vectors such as lentiviral or retroviral vectors encoding for a CAR receptor. This modification normally has no significant effect on cellular metabolism or mitochondrial dynamics. Once the genetically modified CAR T cells are produced, they enter a stage of proliferation in which they further multiply their mitochondria and continue to rely on glycolysis to meet their increased energy demands. Subsequent steps include purification and quality control, culminating in the production of the infusion product, which consists mainly of effector T cells (Teff), effector memory T cells (Tem), and central memory T cells (Tcm). Effector T cells are characterized by a high rate of glycolysis and increased mitochondrial biomass, which enables the cells to respond rapidly to target cells and effectively perform their cytotoxic function within a short period of time. Central memory T cells, on the other hand, typically possess elongated mitochondrial structures and primarily utilize oxidative phosphorylation, allowing them to extend their lifespan and persist in the organism. The phenotype of effector memory T cells can be simplistically viewed as a combination of both and therefore exhibits both glycolytic and oxidative phosphorylating metabolism. Once infused into the patient, CAR T cells rapidly recognize tumor cells and exert a cytotoxic effect on them. Cytotoxicity is a highly energy-consuming process characterized by a high rate of glycolysis and increased mitochondrial biomass. Mitochondria are polarized along the cellular cytoskeleton toward the immunological synapse, providing the energy required for production, polarization, and formation of the immunological synapse, as well as for transfer of lytic granules into target cells to induce apoptosis - in the case of CAR T cells, apoptosis of target cancer cells. Remarkably, a single CAR T cell can eliminate multiple cancer cells. Following the cytotoxic effect, the majority of T cells become exhausted, with mitochondria undergoing mitophagy and the cells losing their effector function as all types of metabolism diminish. These cells may undergo apoptosis, initiated by the mitochondria, leading to rupture of cell structures and cell death. However, a small subset of cytotoxic cells transforms into central memory T cells, forming a permanent immunological memory for the specific antigen.

To better understand the state of immune cells during the process of CAR T cell therapy, it is important to note that at the time of leukapheresis, the patient’s T lymphocytes have usually already undergone at least two lines of other cancer therapies (9). These cells, influenced by the previous lines of immunosuppressive medication and the inhibitory tumor microenvironment (persistent antigen stimulation, inhibitory signaling, hypoxia, acidosis, etc.), often enter the CAR T production process already exhausted, terminally differentiated, and with impaired mitochondrial function (54, 55). During the production process, the cells are activated, genetically modified, proliferated, kept, and stored in *in vitro* conditions (56, 57). Upon infusion into the patient, it is desired that the CAR T cells further clonally expand, migrate rapidly to the tumor site, recognize, and efficiently kill tumor cells, with each CAR T cell eliminating as many tumor cells as possible (58, 59). Since all of these processes are extremely energy consuming, adequate energy production and cellular energy metabolism are crucial for an effective and successful therapy course. In this context, mitochondria play a key role as cellular organelles, responsible for energy production and metabolism (60, 61), constantly adapting to environmental stimuli and the energy demands of the cell. A simplified schematization of mitochondrial dynamics during different phases of CAR T cell therapy is presented in **Figure 1**.

For successful therapeutic outcomes at each phase of the process, it is imperative that mitochondrial function remains robust and demonstrates rapid adaptability to alterations in the cellular milieu and metabolic demands. Five main groups of mitochondrial processes and their potential impact on CAR T cell therapy are further discussed. These are metabolic reprogramming, mitochondrial mass and biogenesis, mitochondrial membrane potential, production and neutralization of reactive oxygen species (ROS), and mitophagy.

Metabolic reprogramming in T lymphocytes refers to the shift in cellular metabolic pathways in response to changes in cellular energy requirements. The primary cellular metabolism in naive, non-activated T cells is oxidative phosphorylation, in which ATP is generated by the transfer of electrons through the electron transport chain at the inner mitochondrial membrane, producing few toxic byproducts and efficiently utilizing glucose (62, 63). However, when cells’ energy demands increase (e.g., during activation, proliferation, cytotoxic activity, or other complex cellular processes), cells shift their metabolism toward glycolysis (64). The latter produces ATP molecules faster, but less efficiently and with the production of toxic byproducts, such as excessive lactate, which can lead to acidification of the cellular environment and loss of cellular functions (65). In addition to glucose metabolism, other catabolic pathways, such as efficient fatty acid oxidation, play critical roles in T cell development, central memory differentiation, cell survival, and long-term remission (66). While shifts in metabolic pathways in healthy cells occur regularly in response to stimuli for altered energy demands, it has been shown that the most effective CAR T cells possess a balanced metabolic profile and are characterized by the ability to quickly shift from one metabolic type to another and vice versa. Inefficient metabolic shifts can result in prolonged glycolysis, inefficient energy production, and consequently ineffective and short-lived CAR T cells (60, 67–69).

Adequate mitochondrial mass is another critical factor defining cellular energy production capacity (70). Along with the increased energy demands and metabolic switch to glycolysis in T lymphocytes or CAR T cells, these cells enhance their mitochondrial biogenesis, resulting in elevated number of mitochondria per cell, and increased mitochondrial size and mass to increase the energy production capacity (71). For the CAR T production process, it is desired that the input T cells have intact mitochondrial function and high mitochondrial biomass (67). After T cell selection, the cells first undergo activation characterized by mitochondrial fission and multiplication. This leads to the formation of punctate mitochondria with loose cristae, reducing the efficiency of oxidative phosphorylation and triggering the initiation of glycolytic metabolism characteristic of effector T cells (60, 71). After effector function, a small proportion of T lymphocytes transform into a memory phenotype with large, elongated mitochondria. These mitochondria possess a high capacity for energy production, which enables them to maintain oxidative phosphorylation and allows the cells to persist in the organism for prolonged time periods (60, 70). However, most effector T lymphocytes become exhausted, the mitochondria disintegrate, shrink, and all metabolic types vanish, leading to cell apoptosis (71). In the context of predicting CAR T therapy progression, Rostamian et al. (60) found that impaired mitochondrial function with low mitochondrial biomass prior to infusion of CAR T cell product leads to poor therapeutic outcomes.

The mitochondrial membrane potential (ΔΨm), generated by pumping protons from the mitochondrial matrix into the intermembrane space, is another indicator of mitochondrial function and the antitumor efficacy of CAR T cells and is crucial for efficient ATP synthesis (72). High mitochondrial membrane potential characterizes the effector phenotype of T lymphocytes, along with increased glycolysis, ROS production, and cellular impairment. In contrast, low mitochondrial membrane potential is characteristic of naive and memory T lymphocytes and is favored in input cells in the CAR T production process for better energy production capacity of the final product (60, 68). In terms of cytotoxic T lymphocytes, lower ΔΨm levels are desirable, as they indicate a better metabolic capacity of the cells, less exhaustion, correspondingly low glycolysis levels, better persistence *in vivo*, better migratory capacity, and antitumor efficacy (68).

Reactive oxygen species (ROS) are oxygen-containing molecules that are mainly generated in the mitochondria (73). Due to their instability, they react rapidly, causing cellular defects at the DNA, RNA, or cellular structure levels, and can even induce cell death (61). Small amounts of ROS are continuously produced and act as signaling molecules, which are then neutralized by cellular antioxidant mechanisms (74). However, under pathological conditions (e.g., cancer) and in exhausted cells, ROS concentrations can greatly increase (71, 75) and damage cellular structures to the point of irreparability (71), impair T cells function (76), and induce T cells senescence (77). Elevated ROS concentrations and impaired antioxidant mechanisms for ROS neutralization in T lymphocytes and CAR T cells prior to infusion of CAR T cell product are indicative of a poor prognosis for effector cell function upon infusion into the patient (60).

Mitophagy is a multistep process that involves recognition of damaged or dysfunctional mitochondria, their uptake into autophagosomes, and subsequent degradation by fusion with lysosomes (78). The process is tightly regulated at multiple levels, including activation of specific mitophagy receptors, recruitment of autophagic machinery components, and coordination of autophagosome-lysosome fusion (79). Mitophagy is essential for proper mitochondrial function in CAR T cells, as it helps to prevent the accumulation of damaged mitochondria that otherwise accumulate excessive amounts of ROS and impair energy production throughout the CAR T production process as well as the therapy course (67, 80).

Mitochondria therefore hold great potential as therapeutic targets to aid the antitumor therapies and as predictive biomarkers for assessing the therapy course prior to CAR T cell infusion (60, 61, 64, 67, 75, 80).

As research continues to illuminate the dynamic role of mitochondria in CAR T cell therapy, understanding and monitoring mitochondrial processes may lead to more effective therapeutic outcomes. Methods to assess mitochondrial function can be categorized at the genomic, transcriptomic, proteomic, and metabolomic levels (81). Primary mitochondrial genetic disorders arise from cellular or mitochondrial pathological mutations (81) that can be identified by genome sequencing analyses (82). At the transcriptomic level, gene expression can be assessed using techniques such as RNA sequencing, polymerase chain reaction (PCR), Northern blotting, microarrays, and many others (83). Epigenetic regulation and post-translational modifications also play an important role in modulating mitochondrial dynamics (84). A variety of techniques are available for proteomic analysis. For example, fluorescently labeled dyes can be used to stain target molecules, allowing determination of their concentration, localization, and dynamics. Such measurements can be performed with fluorescence microscopy (i.e., flow cytometry) and allow visualization and quantification of mitochondrial membrane potential, mass, and other parameters (85). Other common methods for analyzing protein content include Western blotting, electrophoresis, ELISA, chromatography, mass spectrometry, protein microarrays, etc. (81, 83, 84) The Seahorse analyzer is an excellent tool for determining the metabolic status of target cells (86). In addition, high-resolution respirometry, isotope tracking, and other methods have proven useful in this field (87). Other microscopy techniques, such as transmission electron microscopy (TEM), provide high-resolution images of mitochondria that allow direct observation of changes in mitochondrial morphology and structure (88). The analysis of mitochondrial characteristics offers an insight into the cell’s functional state, potentially serving as a biomarker for predicting cell behavior and progression during the CAR T production process. Since mitochondrial characteristics are indicative of cells’ energy capacity, apoptotic susceptibility, and cytotoxic functionality, they could be utilized to forecast the anti-tumor cytotoxicity of CAR T cells before therapy initiation. This prospective approach may allow for the early identification of therapeutic potential of CAR T cells, enhancing patient-specific treatment strategy.

### 2.2 Endothelial activation

**Figure 2:**
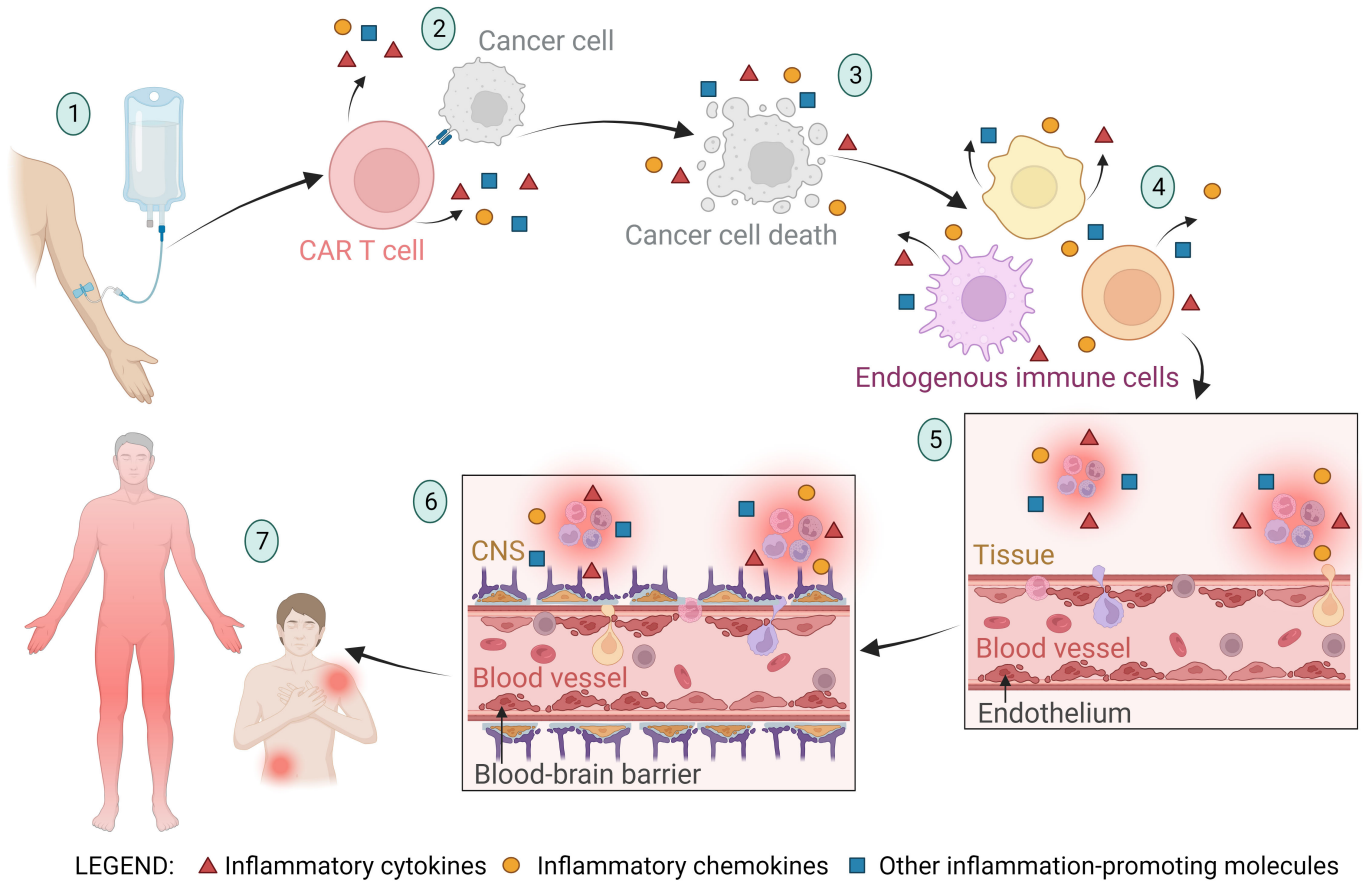
A schematic representation of the stages of endothelial activation and blood-brain barrier permeabilization in CAR T cell therapy leading to the occurrence of cytokine release syndrome (CRS) and immune effector cell-associated neurotoxicity syndrome (ICANS). (1) The CAR T cell product is infused into the patient, and the CAR T cells migrate to the tumor site. (2) CAR T cells recognize the tumor cells and exert a cytotoxic effect on them, triggering the release of inflammatory molecules. (3) Apoptosis and pyroptosis of tumor cells lead to tumor cell death and release of large amounts of cellular components and apoptotic bodies into the bloodstream. The byproducts of tumor cell death trigger the activation of neighboring cells and further stimulate the secretion of inflammatory molecules. (4) The inflammatory molecules from the previously described stages of the CAR T therapy process cause activation of endogenous immune cells (such as macrophages, dendritic cells, neutrophils, natural killer cells, healthy B cells, T cells, and others), resulting in further secretion of inflammatory molecules. (5) Cytokines (primarily IL-6) and other inflammatory molecules stimulate activation and permeabilization of the endothelium, leading to migration of immune cells into the tissue and initiation of inflammation. Activated endothelial cells also begin to secrete inflammatory molecules (such as IL-6), further promoting endothelial activation. (6) Along with endothelial activation, the blood-brain barrier (BBB) is also activated and its integrity is compromised. This allows immune cells and inflammatory molecules to enter the central nervous system (CNS), culminating in CNS inflammation and subsequently the onset of immune effector cell-associated neurotoxicity syndrome (ICANS). (7) Endothelial activation and increased permeability allow immune cells and inflammatory molecules to infiltrate tissues and cause local or systemic inflammation, characteristic of CRS.

The endothelium is a layer of endothelial cells that form the inner lining of blood and lymphatic vessels and play a crucial role in many bodily functions, including the regulation of inflammation, blood clotting, and the formation of new blood vessels (angiogenesis) (89). Upon infusion of CAR T cells, the infused cells migrate to the tumor site and induce apoptosis of tumor cells. In addition to the cytotoxic effect, they secrete cytokines that trigger inflammation and activation of endogenous immune cells (such as macrophages, dendritic cells, natural killer cells, B cells, etc.), fibroblasts, and endothelial cells (90–94). Activated endogenous cells also secrete proinflammatory cytokines and chemokines, which can lead to overactivation of the immune system, endothelial damage, and increased vascular permeability (5, 95). Among these inflammatory cytokines and chemokines, IL-6 is considered the critical cytokine involved in endothelial permeabilization and induction of CRS (5, 96, 97). After endothelial activation and permeabilization, activated endothelial cells also begin to secrete inflammatory signals (such as IL-6). This further leads to increased permeability of the blood-brain barrier, infiltration of inflammatory molecules and immune cells into the central nervous system, and onset of ICANS symptoms such as headache, nausea, confusion, blurred vision, delirium, coma, or even death (98, 99). IL-6 antagonists (such as tocilizumab) are used as intervention drugs to treat severe CRS and ICANS symptoms (96, 97, 100). The stages of endothelial activation and blood-brain barrier permeabilization in CAR T cell therapy leading to the occurrence of CRS and ICANS are shown in **Figure 2**.

In CAR T cell therapy, endothelial activation plays a crucial role in inflammation, regulation of the immune response, and development of side effects (CRS, ICANS, etc.) (5, 101). After administration of the cell product and migration of CAR T cells to the tumor site, the antitumor immune response is initiated. At this time, it is desired that endothelial activation and vascular permeability remain low to moderate to allow for an effective immune response and inflammatory signaling without causing severe inflammation or vascular injury (5). It is important to note that increased endothelial activation and vascular permeability can lead to severe inflammation and high-grade side effects, resulting in less efficient tumor cell killing, unsuccessful therapeutic outcomes, and unmanageable development of side effects that can result in long-lasting consequences and even death (101). Not only does the endothelial activation play a crucial role after administration of the cell product and therapy progression, but studies have also shown that endothelial activation prior to CAR T cell infusion may also contribute to therapy progression and have a negative prognostic effect on CAR T therapy outcome and the occurrence of CRS and ICANS (6). There are many reasons for endothelial activation prior to CAR T cell infusion. On the one hand, it may be a consequence of previous cancer therapies (chemo- or immunotherapy) and the lymphodepleting regimen. Tumor burden with inhibitory tumor microenvironment (TME), hypoxia, and permanent antigen stimulation may also trigger endothelial activation. On the other hand, factors may be un-related to the tumor, such as other medical conditions (diabetes, hypertension, etc.), infections and inflammations, or the physiological state of the patient (obesity, physical performance, age, stress, injuries, etc.) (55, 98, 102). It is usually impossible to select a single factor, but a combination of the listed reasons typically results in excessive activation of the endothelium.

In the context of predicting the outcome of CAR T cell therapy, the Endothelial Activation and Stress Index (EASIX) score has been proposed. It is defined as (creatinine [mg/dL] × lactate dehydrogenase [LDH; U/L])/platelets [10^9^ cells/L] or modified EASIX score combined with CRP × ferritin (EASIX-FC) (42, 43, 103). The correlation between the EASIX score prior to CAR T cell infusion and the occurrence of CRS and ICANS was confirmed (44, 103). However, while EASIX can be a useful predictive tool, it does not directly measure endothelial activation. Instead, it uses surrogate biomarkers that may be influenced by various other factors and accompanying pathological conditions.

In the search for better biomarkers of endothelial activation, the candidates can be classified into three groups based on their effect on the endothelium. The first are endothelial stabilizers, which are mainly synthesized by the endothelium and released into the bloodstream. They are responsible for maintaining endothelial homeostasis and stability and are absent or under-expressed in pathological conditions with endothelial overactivation. Common examples of endothelial stabilizers are nitric oxide (NO) (104), VE-cadherin (105), antioxidant compounds such as superoxide dismutase (SOD) and catalase to combat oxidative stress and maintain endothelial stability (106), extracellular matrix (ECM) components such as collagens, laminins, fibronectins, etc., that structurally support the endothelium and are responsible for maintaining endothelial barrier function (107), and many others. The next group of biomarkers for endothelial activation are endothelial destabilizers, which can be secreted from various cell types and are typically elevated in pathological conditions such as inflammation, stress, cancer, injury, and others. Some common examples of endothelial destabilizers are inflammatory cytokines, such as tumor necrosis factor-alpha (TNF-α) and IL-6 (108, 109), ROS that can cause oxidative stress and damage to the endothelium (110, 111), matrix metalloproteinases (MMPs) that can degrade the ECM and impair endothelial structural support and barrier function (107, 108), and many others. The next important group of endothelial activation biomarkers are endothelial adhesion molecules, which are expressed on the surface of endothelial and other cells and play a crucial role in the interaction and adhesion of leukocytes, other cells, and the ECM to the endothelium (112, 113). Endothelial adhesion molecules play dual roles in stabilizing and destabilizing the endothelium and also in controlling the contact between CAR T cells and their targets (114). Under normal physiological conditions, they contribute to the maintenance of vascular integrity and homeostasis by regulating leukocyte recruitment and transendothelial migration. However, in pathological conditions such as inflammation, infection, or cancer, excessive or prolonged expression of adhesion molecules can lead to endothelial destabilization, increased vascular permeability, and leukocyte infiltration (114, 115). To highlight only a few of the important examples of adhesion molecules, Intercellular Adhesion Molecule-1 (ICAM-1), angiopoietin-2 (Ang-2), Vascular Cell Adhesion Molecule-1 (VCAM-1), and others contribute to endothelial permeabilization. In elevated concentrations, they exhibit a poor prognostic effect and may immunosuppress CAR T cells (116, 117).

It is important to note that to maintain endothelial homeostasis, a precise balance between stabilizing and destabilizing signals must be maintained. Many of the endothelium-related molecules are released into the circulation and can be easily measured from blood samples. They therefore represent a great source of potential biomarkers for predicting CAR T cell therapy outcomes and side effects susceptibility. Moreover, endothelial markers, reflecting the state of vascular health, could serve as valuable tools for predicting therapeutic outcomes even before the initiation of CAR T cell therapy process. Their role in vascular integrity and reaction to inflammatory stimuli makes them promising indicators for assessing the efficacy and potential side effects of treatments in advance.

### 2.3 Central nervous system impairment

The term central nervous system (CNS) includes the brain, spinal cord, nerves, and associated cells. The causes of CNS impairment and injury may be due to concomitant diseases and disorders (autoimmune diseases, neurodegenerative diseases, stroke, etc.), neurological diseases of exogenous origin (toxins, inflammation, infection, injury, etc.), or tumor burden and the TME. The malignancy itself can promote inflammation, tissue damage, and CNS impairment, but prior cancer therapies (chemo- or immunotherapy) and the lymphodepleting regimen may also have an impact (6, 9, 118, 119).

The connection between CNS impairment and the outcome of CAR T cell therapy is an emerging area of research. Focusing on even the smallest changes in markers of blood-brain barrier disruption and markers of neuronal and glial injury could help in predicting and monitoring the progression of ICANS (6, 119–121). Schoeberl et al. (122) observed that efficient ICANS prediction could be achieved in patients without a history of neurological disorders, while patients with accompanying neurological disorders and diseases show signs of previous and/or chronic neuronal damage and respond very heterogeneously to the treatment. Therefore, the predictive accuracy for therapy outcomes and ICANS is limited to individuals without prior neuronal injuries (122).

Biomarkers for determining CNS impairment can be monitored after cell infusion to observe disruptions in CNS homeostasis. The measured values can serve as early indicators of ICANS. However, an emerging field is the use of biomarkers of CNS impairment prior to infusion of CAR T cells. These markers reflect impaired CNS homeostasis and possible CNS injury that may later lead to the development of high-grade ICANS (118). Recent studies have shown that levels of CNS impairment markers prior to CAR T cell infusion correlate with the development of ICANS after CAR T cells administration (121, 123, 124). Several notable biomarkers of neuronal or glial injury have been identified that show considerable promise for predicting the occurrence of ICANS with CAR T cell therapy. Such examples include neurofilament light chain (NfL), a protein originally located in neurons and released into the cerebrospinal fluid (CSF) and into the bloodstream during neuronal injury (118, 122); glial fibrillary acidic protein (GFAP) which indicates astrocyte activation and astrogliosis, often associated with neuroinflammation (123, 124); S100 calcium-binding protein B (S100B), which is released by activated astrocytes and indicates CNS injury (124), and many others. These markers are secreted into the CSF upon CNS injury, but their concentrations in the CSF correlate directly with their concentrations in the blood and can therefore be easily measured from a blood sample (122, 125, 126). Furthermore, the predictive value of CNS impairment markers prior to therapy initiation is gaining attention. By assessing these markers before starting CAR T cell therapy, clinicians might better anticipate therapeutic outcomes and the risk of ICANS, enabling more tailored and proactive management strategies. This approach leverages the correlation between pre-treatment levels of CNS markers and the likelihood of subsequent ICANS, highlighting their utility in enhancing patient-specific therapeutic strategies.

### 2.4 Markers of the immune system

The concept of “immune system markers” encompasses diverse facets of a patient’s heterogeneous immune system. Such aspects include the patient’s baseline characteristics such as age, performance status, organ function, comorbidities, immune system characteristics, immune cells function and exhaustion, and other factors. These characteristics may influence the course and outcome of CAR T cell therapy (37, 45, 47, 55, 102, 127, 128). Moreover, immune system function markers may denote cell markers that differentiate between subpopulations of immune cells and define their phenotypic characteristics (47, 129). This has notable implications for the production process of CAR T cells and their subsequent antitumor efficacy post-administration (47, 55). Therefore, an in-depth understanding of individual immune systems and immune cells characteristics could potentially pave the way for improved prediction of response to CAR T cell therapy and resulting therapeutic outcomes.

Various baseline characteristics and blood biomarkers have been identified that might predict the outcomes of CAR T cell therapy, thus highlighting their importance for therapy selection and management. Among physiological measures, parameters such as age, heart rate, body temperature, comorbidities, and blood pressure have displayed the highest predictive values (47, 128, 130). Among blood biomarkers, leukocyte count, inflammatory cytokines, hemoglobin, creatinine, CRP, ferritin, fibrinogen, and platelets have been shown to predict the development of severe CRS (29, 52, 131, 132). However, consideration of these patient characteristics alone does not provide a sufficiently specific and robust predictive model for application in a broader population. Given their high variability, which may be influenced by previous therapies, patient lifestyle, and disease burden, baseline patient characteristics should be used in conjunction with more robust biomarker systems (47, 55).

Given the nature of CAR T as a T cell therapy, T cell biomarkers are frequently being monitored throughout the process. For instance, studies have shown that a defined CD4:CD8 ratio of T lymphocytes at the time of leukapheresis (ranging from 1:1 to 3:1) is associated with a better proliferative capacity for the CAR T production process (129, 133, 134). A higher CD45RA : CD45RO ratio at the time of leukapheresis indicates an increased proportion of naive, less differentiated T cells correlated with improved proliferative capacity and therapeutic outcome (67). CAR T cell subsets can be distinguished as naive T cells (CD45RO−/CD62L+/CD27+), central memory T cells (CD45RO+/CD62L+/CD27+), effector memory T cells (CD45RO+/CD62L−/CD27−), and effector T cells (CD45RO+/CD62L−/CD27−). Activated CAR T cells express activation markers such as CD25, CD69, and CD137 (94, 135, 136). Furthermore, studies indicate that higher levels of central memory T cells (Tcm) and lower levels of effector T cells (Tef) in the infusion product are associated with improved therapeutic outcomes (50, 94, 129, 136, 137). Elevated levels of exhausted and senescent (CD57+) T cells in the infusion product correlate with poor therapy progression (94, 138). To achieve long-term remission, it is therefore advantageous to have memory CAR T cells that persist over time and provide an efficient antitumor response in the event of relapse (139). New phenotyping biomarkers with higher predictive capacity for CAR T therapy progression are being extensively investigated.

**Figure 3:**
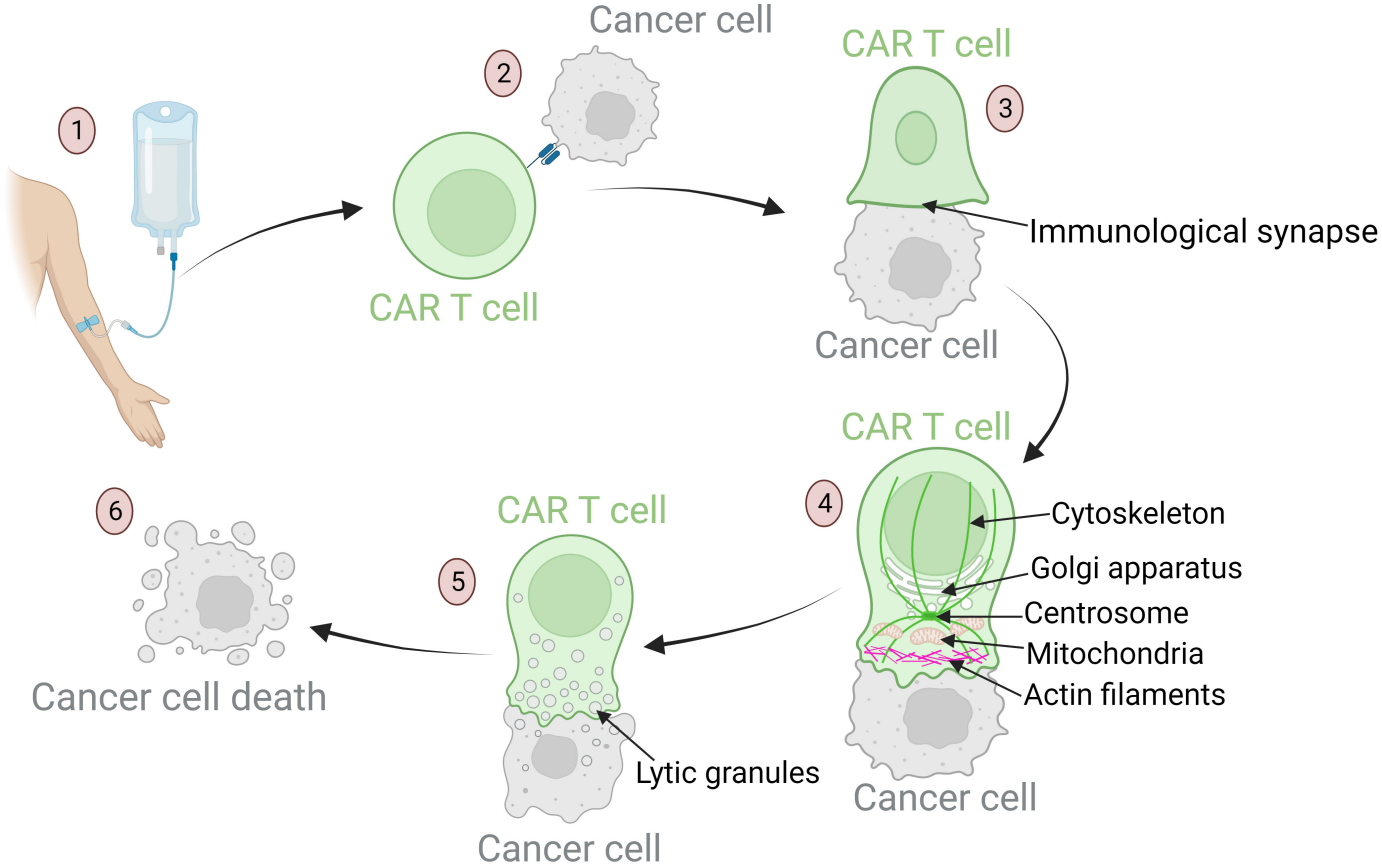
Mechanisms of anti-tumor response of CAR T cells: from target recognition to tumor cell apoptosis. (1) Upon infusion into the patient, CAR T cells migrate through the bloodstream to tumor sites. Their homing ability is influenced by chemokines and adhesion molecules that ensure these cells reach the designated area. (2) Once in the tumor vicinity, CAR T cells recognize specific tumor-associated antigens. This recognition is crucial for precision in targeting. Defects in this process can result in off-tumor toxicities and the development of severe side effects. (3) After recognition of the target antigen, the CAR T cell binds to the tumor cell, leading to cytoskeletal reorganization and formation of the immunological synapse – a specialized interface between the CAR T cell and its target. (4) Dynamic reorganization of CAR T cell components is required to achieve a cytotoxic effect. The cellular organelles responsible for cytotoxic processes travel along the reorganizing cytoskeleton toward the immunological synapse: actin filaments provide structural support for the lamellipodium near the synapse; the centrosome guides cytoskeletal reorganization; the Golgi apparatus aids in the formation of cytotoxic vesicles; mitochondria provide the energy required for cytotoxic processes. (5) These mechanisms culminate in the formation of lytic granules. These granules are transported into the target cell through the immunological synapse. (6) Lytic granules induce apoptosis of tumor cells.

Another significant component of biomarkers of the immune system pertains to the antitumor cytotoxic activity of the CAR T cells. The mechanisms entailing migration, tumor cell recognition, cytokine release, and target cell killing are highly complex and play key roles in successful antitumor efficacy of CAR T cells after administration of the cell product into the patient (29, 114, 140, 141). The specific steps of the cytotoxic function are shown in **Figure 3**. Any malfunctions within these cytotoxic mechanisms can lead to unsuccessful therapy and severe inflammation. Such malfunctions may stem from relatively rare genetic disorders or from T cells dysfunction, which may be a consequence of disease burden, patient characteristics, and exhausted and senescent T cell phenotypes prior to the CAR T production process (29, 55, 102, 142). The malfunctions may also arise during the production process, as cells respond differently to *ex vivo* manipulation due to their individual characteristics (55). The vector encoding the CAR receptor is integrated semi-randomly into the genome, leading to variable expression and consequently variable efficacy of the CAR T cells. The receptors can also be expressed constitutively for extended periods or inductively for a brief duration (143, 144).

Furthermore, the integration of transgenes via viral vectors raises concerns about potential risks, including insertional oncogenesis, gene inactivation or dysregulation, and impairment of cell functions (144). Early detection of such integration events is crucial for ensuring the safety of CAR T cell therapies. Potential biomarkers, such as abnormal gene expression levels, novel fusion transcripts, epigenetic changes, etc., and assays, such as linear amplification-mediated PCR (LAM-PCR), high-throughput sequencing (i.e. integration site sequencing), whole-genome sequencing (WGS), etc. could serve to identify transgene integration sites (145). Advanced bioinformatics tools are further used to analyze the data to assess the potential impact of transgene integration on gene expression (145). Monitoring these integration events could provide insights into the safety profile of CAR T cell products and help mitigate risks associated with gene therapy.

After infusion of the CAR T cell product, the modified cells first migrate to the tumor site. The migration and infiltration into tumor tissue are the main obstacles of CAR T efficiency in solid tumors (146) but also play an important role in hematologic malignancies (99, 115). Adequate expression of adhesion molecules (e.g., LFA-1, VCAM-1, ICAM-1, VEGFA, and others), chemokines (e.g., CCL3, CCL4, CXCL9, CXCL10, CXCL11, CXCL12, and others), and other guidance molecules are of paramount importance for effective CAR T cell homing (114, 147–153). Therefore, inadequate expression of these navigation-related molecules could serve as a negative predictive factor for the progression and outcome of CAR T cell therapy (114, 149, 150). For successful effector functions, it is imperative for CAR T cells to rapidly recognize tumor cells and facilitate CAR receptor binding with the CAR antigen (e.g., CD19, CD20, CD22, BCMA, etc.) expressed on tumor cells (154). Recent clinical evidence indicates that antigen downregulation and escape have arisen as major obstacles that affect the overall efficacy, success rate, and long-term remission after CAR T cell therapy (155). Therefore, sufficient antigen expression on tumor cells may serve as a prognostic tool for therapy outcomes and may even influence patient eligibility for CAR T cell therapy. Upon recognition of target cells, CAR T cells trigger a series of cytotoxic reactions aimed at inducing target cells apoptosis.

In the event of abnormalities within cytotoxic mechanisms or prolonged duration of the immunological synapses leading to an extended effector function timeframe, this could escalate the release of cytokines and chemokines, thus increasing inflammation, compromising the efficacy of the therapeutic response, and potentially causing a relapse of antigen-free malignancy (29, 140, 142, 147, 156–158). Methods for *ex vivo* examination of cytotoxic efficiency of CAR T cells are described in **Table 2**. A careful examination of cytotoxic mechanisms of T cells prior to CAR T cell infusion may provide insight into potential defects and serve as an initial indication of therapy prognosis and the probability of severe inflammation occurrence. This information is crucial for predicting the course of CAR T cell therapy prior to cell infusion.

**Table 2:** Methods for *ex vivo* investigation of the cytotoxic efficacy of CAR T cells.

Technique	Methodology	Indices	Ref.
Co-culture of CAR T cells with fluorescently-labeled tumor cells	Fluorescence microscopy	Decrease in fluorescence indicates tumor cell lysis	(147)
Chromium release assay	Detection of released radioactive chromium isotope from target cells	Elevated levels of released chromium isotope indicate higher level of target cell apoptosis	(156)
LDH release assay	Colorimetric assay	Elevated levels of released LDH indicate higher level of target cell lysis	(157)
Release of effector cytokines	ELISA	Sufficient levels of effector cytokines are released during successful target cell killing	(139, 141)
Release of degranulation markers	Flow cytometry	Effective release of cytotoxic granules leads to effective target cell killing	(141)
Expression of cytotoxicity-related proteins	qPCR	Elevated levels indicate better cytotoxic reactivity	(29)
Real-time impedance-based assays	Electrical impedance measurements	Monitoring cellular interactions, cytotoxicity, and cell lysis	(158)
Tumor spheroids or organoids	Modeling tumor cell killing	Tumor spheroids or organoids can serve as targets of tumor cell killing	(139)
Multiparametric flow cytometry	Flow cytometry	evaluation of different markers of activation, exhaustion, and cytotoxicity	(156)
Time-lapse microscopy	Live-cell imaging platforms	Visualizing CAR T cell interactions with tumor cells and monitoring tumor cell elimination kinetics	(140)
Polyfunctionality measurement	Various methodologies assessing multiple functions simultaneously (cytokine production, proliferation, target cell killing, etc.)	A more comprehensive indication of CAR T cell cytotoxic efficacy	(159)

### 2.5 Extracellular vesicles

Extracellular vesicles (EVs) are small membrane-derived particles that are released by cells into the extracellular space and can be transported throughout the body. These vesicles play an important role in cell-to-cell communication and transport a variety of biological molecules from their cell of origin to target cells (160, 161). Because they are derived from parent cells, the EVs carry markers of parent cell that allow the origin of the vesicles and their contents to be determined (162). By analyzing the vesicles content, cellular signaling can be monitored, providing insight into cell-to-cell communications (163). Their usual cargo is proteins, lipids, DNA, messenger RNAs (mRNAs), microRNAs (miRNAs), and other molecules (164).

Because EVs are involved in many physiological and pathological processes, their content provides valuable insights into the signaling of specific cell populations. For example, they can mediate immune responses, facilitate blood clotting, and contribute to the spread of cancer (160, 165, 166). In the case of CAR T immunotherapy, this could prove useful in assessing the immune system status, immune cell exhaustion and functionality, and antitumor response. On the other hand, by studying tumor cell-derived extracellular vesicles (oncosomes), the information on tumor invasiveness, antigen escape, and inhibitory signaling toward cells of the immune system, including CAR T cells, could be better understood (163, 167). EVs were shown to exhibit an effect on CAR T cells (168, 169). Due to their ability to transport molecules from one cell to another, EVs are being extensively studied for their potential use as drug delivery systems and as biomarkers for disease prognosis and immunotherapy progression (170–172).

To discuss some examples of EVs that could potentially predict response to CAR T cell therapy and the development of high-grade CRS and ICANS, the origin of the vesicles must be taken into consideration. First, the vesicles can be derived from endogenous immune cells. They can exhibit stimulatory or inhibitory functions toward CAR T cells and therapy response (173, 174). For example, an increased number of CD69 positive T cell vesicles can indicate increased T cell activation and act as a negative feedback loop that inhibits further T cell activation (175). Increased numbers of T cell EVs expressing inhibitory molecules such as PD-1, CTLA-4, TIM-3, LAG-3, and others reflect an ineffective and exhausted immune system and could consequently be used to predict poor response to therapy (169, 174, 176).

Possible sources of EVs are also CAR T cells. Studies have shown that persistent concentrations of CAR-positive EVs in the bloodstream of patients after CAR T cell infusion exhibit predictive impact on long-lasting remission (177). Evidence also suggests that CAR-positive EVs assist the antitumor function of CAR T cells by overcoming obstacles and barriers that otherwise limit the effect of the immunotherapy (178–180). The next example is increased levels of endothelial vesicles and apoptotic bodies, which indicate excessive endothelial activation and damage, which may predict the development of severe CRS and ICANS even before infusion of the cell product (170). Tumor-derived vesicles often express inhibitory molecules and reduce the antitumor effect of CAR T cells (169). Elevated levels of circulating tumor DNA correlate with poor therapeutic efficacy and higher CRS levels (181), which can also be applied to circulating oncosomes containing tumor DNA. CD19+ vesicles were shown to cause activation and exhaustion of CAR T cells with decreased antitumor activity and trigger CRS (175).

Stated here are just some common examples of EVs and their potential impact on immunotherapy. The EVs show great potential for predicting the immune response to CAR T cell therapy. However, this field is relatively young and poses many challenges. The first is the development of standardized and optimized extraction procedures for isolation of heterogeneous vesicles from cancer patients’ samples. Because vesicles vary in density, structure, and size, robust isolation techniques with minimal sample loss need to be established (165). Another challenge currently being investigated by many research groups is the development of biomarkers to efficiently differentiate B-cell leukemia or lymphoma from other types of vesicles. Some studies suggest examples such as CD5, CD19, CD31, CD44, CD55, CD62L, CD82, and CD123 (169, 182, 183). Further research on this topic is needed to develop efficient biomarkers and predictive models. EVs could serve as predictors before CAR T cell infusion to provide an impression of cellular signaling and information circulating in the patient’s bloodstream.

### 2.6 Inhibitory tumor microenvironment

The tumor microenvironment (TME) is a complex mixture of various components, including different cell types, signaling molecules, and extracellular matrix components. The TME can contribute to tumor growth, progression, and inhibition of the antitumor immune responses (184, 185). Consequently, the TME components may undermine the cytotoxic potency of CAR T cells, thereby limiting the efficacy of CAR T cell therapy. Accordingly, assessment of individual patient TME characteristics and the inhibitory properties of the TME components on CAR T cells prior to CAR T cell infusion may serve as predictive parameters for determining the potential extent of CAR T cell effector function inhibition after infusion. This could allow the prediction of inflammation development associated with immune cell inhibition.

**Figure 4:**
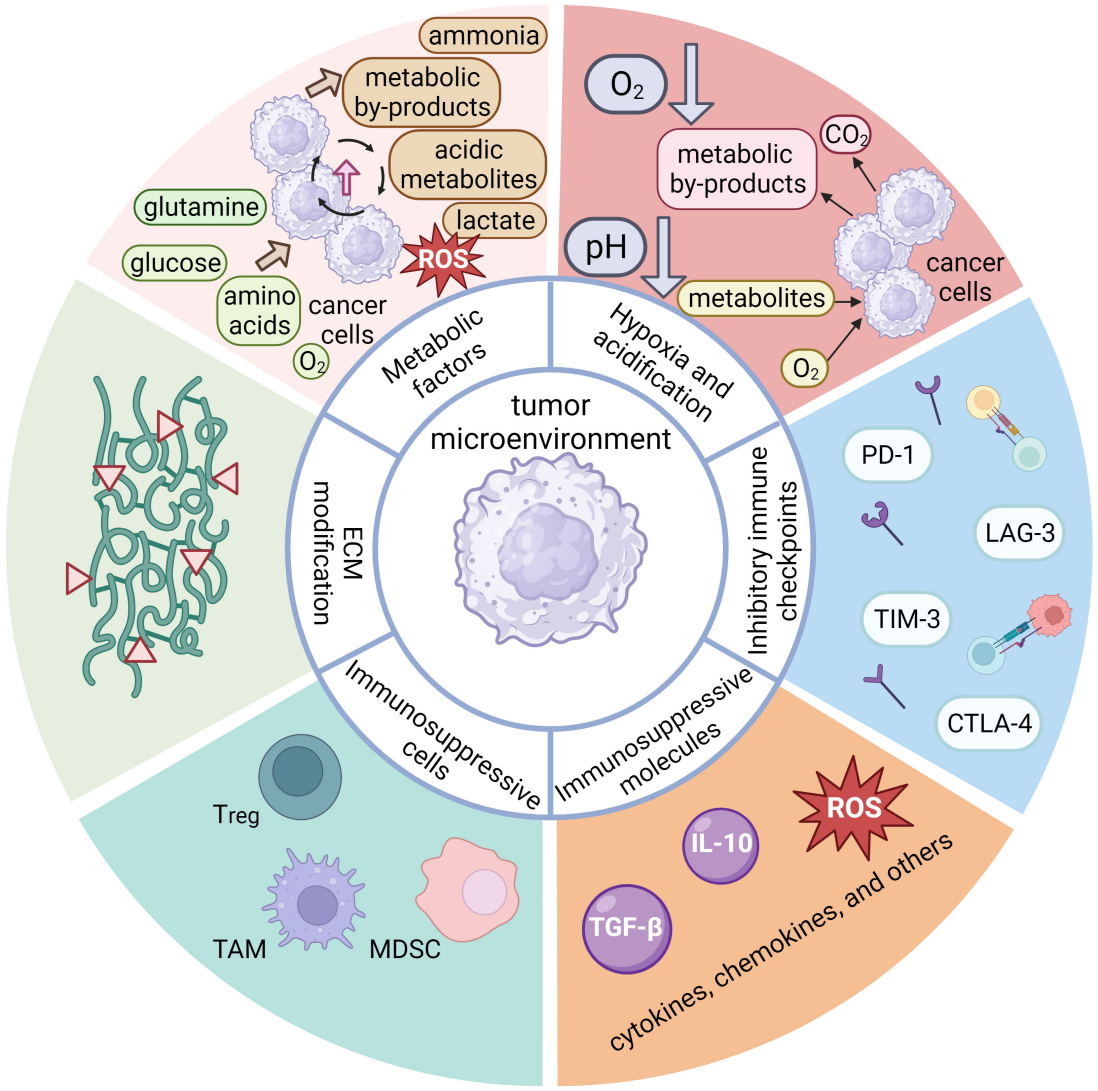
A schematic representation of the main components of the inhibitory tumor microenvironment (TME) associated with hematologic malignancies, which possess inhibitory properties toward the anti-tumor response of the immune system. The components of hematologic TME can be divided into six main groups. One important component are the immunosuppressive cells, which include regulatory T cells (Tregs), tumor-associated macrophages (TAMs), myeloid-derived suppressor cells (MDSCs), tumor cells, and others. These immunosuppressive cells can secrete immunosuppressive molecules, along with other patient cells. These molecules are cytokines (IL-10, TGF-β, etc.), chemokines, or others (e.g., reactive oxygen species), all of which may exert an inhibitory effect on the anti-tumor functions of the immune system. The expression of inhibitory immune checkpoint molecules (PD-1, CTLA-4, TIM-3, LAG-3, etc.) is another important aspect of inhibitory TME that leads to cellular exhaustion and ineffectiveness of immune cells. Although hypoxia is more characteristic of solid tumors due to poor perfusion of tumor tissue and metabolic processes, it also plays an important role in hematologic diseases. Its effect is more pronounced in the bone marrow and can lead to accumulation of immunosuppressive cells and molecules that inhibit the effector function of T cells. Tumor cells are characterized by enhanced metabolic processes leading to excessive uptake of glucose, glutamine, amino acids and O_2_. The high nutrient uptake by tumor cells can deprive immune cells of nutrients, thereby impairing their metabolic processes and overall fitness. Excessive metabolic byproducts, such as lactate, CO_2_, other acidic metabolites, ammonia, and ROS are also produced and secreted into the TME, often leading to a drop in pH and subsequent immunosuppression. Finally, tumor cells can secrete various enzymes (e.g., metalloproteinases, collagenases, oxidases, etc.) that can degrade or alter the components of the extracellular matrix (ECM), which is critical for maintaining tissue integrity. The thickening and alteration of the ECM can inhibit the anti-tumor immune response, facilitate tumor growth and spread, and promote inflammatory processes.

The TME constituents are divided into six main categories based on their composition and function. First are immunosuppressive cells, which include regulatory T cells (Tregs), myeloid-derived suppressor cells (MDSCs), tumor-associated macrophages (TAMs), and other (113, 186–189). The TME immunosuppressive cells can inhibit the antitumor response in several ways: by inducing anergy, exhaustion, or even apoptosis of T cells (190, 191), by inducing expression of immunosuppressive cell markers (such as immune checkpoint molecules) (187, 189, 192), by signaling proliferation and recruitment of other immunosuppressive cells (189, 192), by altering antigen presentation, which impairs recognition of the tumor by the immune system (187), by altering metabolic pathways to deplete energy sources and produce toxic metabolites (190), and by secretion of immunosuppressive molecules such as cytokines, chemokines, and others (187, 188, 192). The TME can induce expression of inhibitory immune checkpoints, typically expressed on various immune cell types. Normally, these molecules regulate and control the immune response to prevent over-activation. However, in the context of cancer, tumor cells can exploit their mechanisms to downregulate the immune system response, thereby facilitating evasion of the immune system. Prominent examples of inhibitory immune checkpoints include PD-1, CTLA-4, TIM-3, LAG-3, etc. (193, 194) Moreover, these inhibitory checkpoint molecules can hinder the activation and functionality of CAR T cells after infusion (193), and their quantitative expression serves as an estimate of their inhibitory effect on CAR T cells (195). Albeit more common in solid tumors, hypoxia may also manifest in the bone marrow microenvironment and contribute to immunosuppression in hematologic malignancies (187, 190). Hypoxic conditions can induce accumulation of immunosuppressive cells and molecules, inhibit the effector function of T cells, and promote immune evasion by tumor cells (113, 196). Tumor cells also expedite metabolic pathways to produce sufficient energy for tumor growth, depriving tissues of nutrients such as glucose, glutamine, amino acids, O_2_, etc. (113, 190, 197) This increased metabolic activity generates toxic or acidic metabolic byproducts (such as lactate) (112, 190) that contribute to acidification of the tumor microenvironment and subsequently suppress the immune response and CAR T cell function (112, 113, 196). The dysregulation of metabolic pathways and the imbalance of metabolites can result in the production of ROS, causing further damage to immune cells and tissues, inhibiting the antitumor effect of T lymphocytes and CAR T cells, and promoting tumor growth (113, 191). Another significant impact of the TME is the degradation and alteration of the ECM by degradative enzymes secreted by tumor cells (e.g., metalloproteinases, collagenases, oxidases) (192, 198). Degradation and alteration of the ECM can lead to impaired tissue integrity, accumulation of metabolic byproducts, and promotion of tumor spread and growth (112, 199). The main components of the inhibitory TME associated with hematologic malignancies are shown in **Figure 4**.

Considering the factors described above, the characteristics of the TME are increasingly recognized as potential predictive biomarkers for CAR T cell therapy progression even before infusion of the cell product into the patient. For instance, the presence of specific immunosuppressive cell types (such as CD4+/CD25+/FOXP3+ Tregs) (99, 195), the expression of certain inhibitory molecules (e.g., PDL-1, TGF-β, IL-10, ROS, etc.) (55, 112, 200), and the overall metabolic state of the TME (e.g., lactate, LDH, etc.) (102, 112, 195) could provide insight into the ability of CAR T cells to function effectively after infusion. A thorough understanding of the interplay between CAR T cells and the TME will also aid in development of strategies to overcome the inhibitory environment. Approaches such as co-administration of immune checkpoint inhibitors, supplementation of cytokines, or genetic modification of CAR T cells to resist the immunosuppressive environment are currently being investigated to increase the efficacy of CAR T cell therapy (112, 174, 195, 196). Given the dynamic nature of the tumor immunological microenvironment, which exhibits variations over time, it is of paramount importance to personalize and monitor immunotherapies to maximize the therapeutic efficacy (196). An overview of common techniques for *ex vivo* biomarker analysis of the discussed biomarkers for predicting treatment response and side effects in CAR T cell therapy is presented in **Table 3**.

**Table 3:** Examples of common techniques for *ex vivo* biomarker analysis for predicting treatment response and side effects in CAR T cell therapy.

Category	Biomarkers	Methodology	Indices	Ref.
Mitochondrial dynamics	Mitochondrial membrane potential	Flow cytometry	Indicator of mitochondrial function	(68, 85)
	Oxidative phosphorylation and glycolysis	Seahorse XF Analyzer	Indicator of cellular respiration and energy metabolism	(67)
	GAPDH and LDHA	PCR	Upregulated expression indicates high levels of glycolysis	(64)
	Visualization of mitochondrial morphology and structure	TEM	Indicator of potential mitochondrial impairment	(88)
	ROS	Mass Spectrometry	Elevated levels indicate poor prognosis for T cell effector function	(67)
Endothelial activation	IL-6	Multiplex Bead Array	Induction of CRS	(96)
	Ang2/Ang1	ELISA	Indication of endothelial stability and function	(117)
	ICAM-1, VCAM-1	PCR	Elevated endothelial expression levels exhibit poor prognostic effect on CAR T cells	(101)
	Endothelial EVs (e.g., CD31-positive EVs)	Flow cytometry	Indication of excessive endothelial activation and damage	(170)
	NO	Spectroscopy	Endothelial stabilizer, which may indicate inflammatory processes	(43, 104)
	von Willebrand Factor (vWF)	Immunoturbidimetry	Elevated blood levels of vWF can indicate endothelial damage or dysfunction	(116)
Central nervous system impairment	GFAP	ELISA	Indicates astrocyte activation and neuroinflammation	(118, 123)
	NfL	Single-molecule array assay	Marker of neuronal injury	(121, 122)
	MMP-9	Multiplex assays	Indicates inflammation and disruption of the blood-brain barrier	(107, 108)
	S100B	Chemiluminescence immunoassay	Indicates astrocyte injury and BBB impairment	(126)
Markers of the immune system	PD-1, CTLA-4, LAG-3, TIM-3	Flow cytometry	T cell exhaustion	(156, 194)
	Target cell death	Impedance-based assays	Effective cytotoxicity of T and CAR T cells	(158)
	CRP	High-sensitivity CRP test	Indicates systemic inflammation, usually leading to development of more severe CRS	(48, 103)
	GZMB, GZMA, and PRF1	PCR	Sufficient expression in T cells for successful induction of target cell apoptosis	(156)
	TGF-β and IL-10	Multiplex Bead Array	Inhibition of T and CAR T cell function	(139)
	CCL3, CCL4, CXCL9, CXCL10, CXCL11	ELISA	Homing of CAR T cells to tumor sites	(150, 152)
	CAR T cells and target cells	Live Cell Imaging	Formation of immunological synapse and release of lytic granules to induce target cell apoptosis	(140, 147)
Monitoring extracellular vesicles	miRNA	RNAseq	Inhibition of T and CAR T cell function	(164)
	CD19-positive Evs	Flow cytometry	Unspecific activation and exhaustion of CAR T cells with reduced antitumor activity and triggering CRS	(173, 175)
	CAR-positive EVs	ELISA	Long-lasting remission	(177)
	Leukemia cells derived EVs	Nanoparticle tracking analysis	Potential inhibition of leukemia-derived vesicles on CAR T cells	(169, 175)
Inhibitory tumor micro-environment	Suppressor cells of the TME	Flow cytometry	Inhibition of T and CAR T cell function and induction of apoptosis	(186)
	PDL-1 expression	PCR	Inhibitory signaling resulting in T and CAR T cell exhaustion	(200)
	Lactate	Lactate Test Strips	Metabolic byproduct of cancer cells with inhibitory properties on T (CAR T) cell effector function	(196)
	Tumor-infiltrating CAR T cells	Immunohistochemistry (IHC)	Infiltration and persistence of CAR T cells indicate better therapy response	(140)
	ROS	Chemiluminescence	High levels indicate less favourable environment for T (CAR T) cell function	(196)

## 3 Conclusions and future directions

The emergence of CAR T cell therapy has ushered in a new era of cancer treatment, offering the potential to overcome many of the limitations associated with conventional therapies. However, better approaches for understanding and predicting the therapy progression are needed. This review emphasizes the multifactorial nature of therapeutic outcomes that extend beyond the CAR T cells themselves to include the intrinsic characteristics of the patient’s immune system and the dynamic interplay with the tumor microenvironment. The findings highlight the complexity and variability of the determinants of therapeutic success and suggest that a shift away from a reductionist approach focusing on single biomarkers toward a more integrative perspective is needed. Here, we propose the use of advanced biomarker models that incorporate various aspects of individual immune characteristics as well as the interplay and signaling between the immune system and the malignancy at both the cellular and systemic levels, as discussed in this review. In this way, predictive models could more accurately reflect the complex interactions that occur within the human body, potentially leading to more precise and robust predictions of therapy outcomes and more personalized therapeutic strategies. In conclusion, to realize the full potential of CAR T cell therapy, a comprehensive understanding of the numerous factors influencing its efficacy is needed. Further investigation of the impact and correlation of the discussed factors with therapy progression may lead toward a more personalized approach, which could offer reduced side effects and hold promise for the future use of this advanced immunotherapy.

## Author contributions

LL: Conceptualization, Visualization, Writing – original draft, Writing – review & editing. LJ: Writing – review & editing. AI: Writing – review & editing. AK: Conceptualization, Visualization, Writing – original draft, Writing – review & editing.
